# The INTERVAL trial to determine whether intervals between blood donations can be safely and acceptably decreased to optimise blood supply: study protocol for a randomised controlled trial

**DOI:** 10.1186/1745-6215-15-363

**Published:** 2014-09-17

**Authors:** Carmel Moore, Jennifer Sambrook, Matthew Walker, Zoe Tolkien, Stephen Kaptoge, David Allen, Susan Mehenny, Jonathan Mant, Emanuele Di Angelantonio, Simon G Thompson, Willem Ouwehand, David J Roberts, John Danesh

**Affiliations:** Department of Public Health and Primary Care, Strangeways Research Laboratory, University of Cambridge, Worts Causeway, Cambridge CB1 8RN UK; Department of Haematology, University of Cambridge, Long Road, Cambridge, CB2 0PT UK; NHS Blood and Transplant - Oxford Centre, Level 2, John Radcliffe Hospital, Headley Way, Oxford OX3 9BQ UK; NHS Blood and Transplant, Longley Lane, Sheffield S5 7JN UK; NHS Blood and Transplant, Long Road, Cambridge, CB2 0PT UK; Radcliffe Department of Medicine, University of Oxford, John Radcliffe Hospital, Headley Way, Oxford, OX3 9DU UK; The INTERVAL trial coordinating centre, Department of Public Health and Primary Care, University of Cambridge, Cambridge, CB1 8RN England

**Keywords:** whole blood donation, randomised controlled trial, donation frequency, blood supply, donor well-being

## Abstract

**Background:**

Ageing populations may demand more blood transfusions, but the blood supply could be limited by difficulties in attracting and retaining a decreasing pool of younger donors. One approach to increase blood supply is to collect blood more frequently from existing donors. If more donations could be safely collected in this manner at marginal cost, then it would be of considerable benefit to blood services. National Health Service (NHS) Blood and Transplant in England currently allows men to donate up to every 12 weeks and women to donate up to every 16 weeks. In contrast, some other European countries allow donations as frequently as every 8 weeks for men and every 10 weeks for women. The primary aim of the INTERVAL trial is to determine whether donation intervals can be safely and acceptably decreased to optimise blood supply whilst maintaining the health of donors.

**Methods/Design:**

INTERVAL is a randomised trial of whole blood donors enrolled from all 25 static centres of NHS Blood and Transplant. Recruitment of about 50,000 male and female donors started in June 2012 and was completed in June 2014. Men have been randomly assigned to standard 12-week versus 10-week versus 8-week inter-donation intervals, while women have been assigned to standard 16-week versus 14-week versus 12-week inter-donation intervals. Sex-specific comparisons will be made by intention-to-treat analysis of outcomes assessed after two years of intervention. The primary outcome is the number of blood donations made. A key secondary outcome is donor quality of life, assessed using the Short Form Health Survey. Additional secondary endpoints include the number of ‘deferrals’ due to low haemoglobin (and other factors), iron status, cognitive function, physical activity, and donor attitudes. A comprehensive health economic analysis will be undertaken.

**Discussion:**

The INTERVAL trial should yield novel information about the effect of inter-donation intervals on blood supply, acceptability, and donors’ physical and mental well-being. The study will generate scientific evidence to help formulate blood collection policies in England and elsewhere.

**Trial registration:**

Current Controlled Trials ISRCTN24760606, 25 January 2012.

## Background

In the long-term, ageing populations may demand more blood transfusions, but the blood supply could be limited by difficulties in attracting and retaining a proportionately smaller and decreasing pool of younger donors. [1, 2]. One approach to increase the blood supply, especially for groups for whom supply tends to be more limited (for example, people who have O negative and B negative blood types), is to collect blood more frequently from existing donors. If more donations could be safely collected in this manner at marginal cost, then it would be of considerable benefit to blood services.

The principal risk of more frequent donation is, however, iron deficiency and a fall in haemoglobin levels [3]. Each blood donation removes about 250 mg of iron from the body. There is considerable individual variation in iron stores, dietary intake, and efficiency of absorption. If iron is not fully replaced, then donors’ iron stores become progressively depleted, leading to iron deficiency or the development of frank iron-deficiency anaemia. Iron deficiency or anaemia may result in adverse health consequences for donors and their temporary rejection from giving further donations or ‘deferral’, due to failure to meet the haemoglobin threshold required for donation. This threshold, together with limits for donation intervals, has been set to minimise iron deficiency in repeat blood donors [4].

In the absence of data from reliable randomised trials to inform policies on inter-donation intervals, the Council of Europe has provided directives for blood collection that have been open to different national interpretations [5] (Table 1). NHS Blood and Transplant (NHSBT), the sole blood provider to the National Health Service (NHS) in England, currently allows men to donate up to once every 12 weeks and women to donate up to once every 16 weeks. In contrast, some other European countries allow donations as frequently as every 8 weeks for men and every 10 weeks for women.

**Table 1:** **Whole blood inter-donation intervals across European blood services (weeks)**

	Men	Women
**England**	12	16
**Austria**	8	10
**Finland**	8	12
**France**	8	12
**Germany**	8	12
**Ireland**	10	10
**Estonia**	10	12
**Netherlands**	10	16
**Denmark**	12	12
**Flanders**	12	12
**Wales**	12	16
**Slovenia**	12	16
**Spain**	12	16
**Scotland**	16	16

As part of their duty of care, blood services screen donors at each visit for haemoglobin levels. Across Europe, minimum thresholds of 13.5 g/dL for men and 12.5 g/dL for women must be met for donation eligibility [6]. In Canada and in the USA, the thresholds are 12.5 g/dL for both men and women [5, 7]. In England, about 3% of donors overall are not eligible to donate because of failure to meet the haemoglobin threshold at screening (unpublished data, NHSBT). Such deferral may demotivate individuals to donate in the future and directly or indirectly increase the cost of collecting blood [8, 9]. Furthermore, some donors may not attend scheduled donation appointments or may self-defer owing to symptoms, which could be due, at least in part, to iron deficiency or anaemia. Therefore, the challenge faced by blood services is to decrease the incidence of deferrals for low haemoglobin whilst increasing blood supplies to meet projections of increasing demand.

We have designed the INTERVAL trial, which is a parallel group, pragmatic randomised trial. INTERVAL has enrolled about 50,000 male and female donors at the 25 static donor centres of NHSBT located throughout England. The trial’s key anticipated contribution will be to determine optimum inter-donation intervals that maximise blood supply and maintain well-being of donors. A subsidiary aim of the INTERVAL trial is to explore suggestions that donation intervals could be tailored to donors’ capacity to donate blood safely. For example, it may be that some donors have a decreased capacity for frequent donation (for example, very lean people and/or women of childbearing age who may be predisposed to anaemia), whereas other donors may be able to donate more frequently (for example, men, heavier donors, those who carry genes predisposing to them to higher iron stores) [10–12].

We confirm that recruitment into the trial was ongoing at the time this manuscript was submitted for publication.

## Methods/Design

### Pilot work preceding this trial

Prior to the initiation of INTERVAL, we conducted the Cambridge CardioResource study [13], a study of 2,500 blood donors from the East Anglia region of the UK in order to demonstrate the feasibility of embedding research protocols within NHSBT’s routine service setting. This study showed that it is possible to recruit and consent donors and collect research samples at NHSBT donation sessions, with 99% of samples being successfully retrieved. It also demonstrated that donors could complete additional research protocols remotely by the internet [14]. The findings of this pilot study have informed the design of INTERVAL.

### Objectives

The dual objectives of INTERVAL are (1) to determine the optimum intervals between donations for men and women that maximise blood supply, maintain well-being of donors, and avoid unacceptably increasing risk of iron deficiency/anaemia and its potential complications, and (2) to explore tailoring of blood donation intervals to donors by their demographic, haematological, genetic and/or lifestyle characteristics.

### Hypothesis

We will test the hypothesis that more frequent blood donation than is currently customary in England will increase the total number of blood units collected over 2 years without adversely affecting the health or well-being of blood donors.

### Design

**Figure 1:**
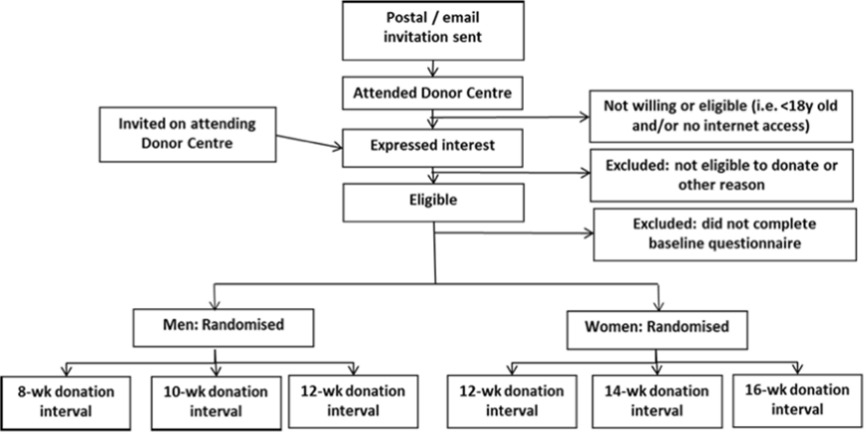
**Overall design for the INTERVAL trial.**

INTERVAL is an open, randomised, multisite trial involving the recruitment of about 50,000 male and female donors from all 25 static donor centres of NHSBT throughout England. Men have been randomly assigned to standard 12-week versus 10-week versus 8-week inter-donation intervals; and women to standard 16-week versus 14-week versus 12-week intervals (Figure 1). To explore the potential value of tailoring donation intervals to possibly relevant subgroups, we have pre-specified analysis of (a) men and women, (b) carriers of informative variants in the human haemochromatosis (HFE) gene, (c) pre- and post-menopausal women, (d) people with body mass index (BMI) <22, (e) new blood donors, and (f) subgroups based on baseline measures of iron status (for example, serum ferritin).

### Setting and participants

NHSBT collects whole blood from donors attending either static donor centres or temporary ‘mobile’ donation sessions set up at community venues such as village halls. Recruitment in INTERVAL has been restricted to donors attending the static donor centres (which are open daily during the working week), principally because ‘mobile’ sessions do not typically visit locations often enough to accommodate donors who would be allocated to the more frequent intervals being evaluated in INTERVAL. The static donor centres of NHSBT are located in Birmingham, Bradford, Brentwood, Bristol, Cambridge, Edgware, Gloucester, Lancaster, Leeds (2 sites) Leicester, Liverpool, Luton, Manchester (2 sites), Newcastle, Nottingham, Oxford, Plymouth, Poole, Sheffield, Southampton, Stoke on Trent, Tooting (South London), and West End London. To facilitate the provision of adequate training support during each site’s first week of participant recruitment, we commenced recruitment in one new centre per week. At each centre, designated trained members of staff adopted the roles of clinical and/or operational experts to supervise the work of the trial.

The overall approach used in INTERVAL has been to embed research activity within the existing operational framework of NHSBT. To support additional functions required in the trial, we have established an academic trial coordinating centre at the Department of Public Health and Primary Care, University of Cambridge. In addition to supporting the trial’s core scientific activities, the coordinating centre provides a helpdesk to respond to queries from participants about the trial, and maintains a study website [15]. The academic coordinating centre has worked closely with the INTERVAL study administration team (ISAT) based within NHSBT. For example, ISAT has supported the trial to enable participants to make appointments to give blood at intervals that are more frequent than current NHSBT practice (which is not possible through NHSBT’s routine appointment system). To enhance adherence of trial participants to their allocated donation intervals, ISAT has used more intensive and systematic efforts than used in routine NHSBT practice to remind participants about their blood donation appointments, including a systematic three-step telephone and email reminder process.

### Participant recruitment

Postal invitations to take part in the trial, which included a copy of the trial information leaflet, have been sent by NHSBT alongside routine invitations to give blood. During the first 6 months of the trial (June 2012 to January 2013), invitations were sent only to those donors who were registered at a static donor centre (approximately 147,000 invitations in total). Between January and October 2013, three additional recruitment strategies were implemented, which included invitations to groups of ‘mobile’ session donors who might be willing to attend a static centre in order to join the trial. These invitations were to donors who (1) had previously indicated a willingness to give platelets at a static centre (approximately 23,000 invitations), (2) gave blood at a ‘mobile’ session within 10 miles of a static centre (approximately 200,000 invitations), and (3) lived within 30 miles (but typically within 20 miles) of a static centre (approximately 189,000 invitations).

Donors received invitations to join the trial at their next scheduled appointment. At that visit, they were asked by the donor centre reception staff whether they had received an invitation for the trial and if they wanted to join. Donors who had not yet received or read any information about the trial but who wanted to join were given the opportunity to read the information leaflet in the waiting area before donating. If there was insufficient time to do this, or the donor wanted more time to think about joining, then a trial information pack was given to the donor to read and consider before their next donation. Donors who had read the information and were willing to take part were asked to confirm their email and telephone contact details.

### Inclusion and exclusion criteria

Participants eligible to take part in the trial are aged 18 years or older, fulfil all normal criteria for blood donation [16], and are willing to be randomised to any of the trial’s intervention groups. In addition, they must be willing to donate at one of the static NHSBT donation centres for the duration of the trial. Study-specific criteria for exclusion from the trial are lack of internet access and/or unwillingness to provide an email address for trial correspondence, since the trial mainly collects data via remote and web-based methods.

### Donor screening and obtaining informed consent

At the donor centres, donors underwent routine screening for donation eligibility, including haemoglobin measurement via a copper sulphate test, followed by a Hemocue™ test for those who failed the copper sulphate test. If the donor was not eligible to make a donation on that day, for any reason, then he/she could not join the trial on that occasion. Donors who were eligible to take part in the trial were asked if they had any further questions, which were answered either directly (aided by ‘frequently asked questions’ material provided by the research team) or forwarded to the trial ‘champion’ at the donor centre or to the trial helpline. At this point, donors were asked to complete and sign both copies of the consent form, which were checked for completion of all relevant sections and for the donor’s signature on both copies. On the ‘trial copy’ of the consent form, the carer affirmed by signature that he/she had witnessed its completion and retained this copy while providing the ‘donor copy’ to the participant. For donors who were not subsequently eligible or willing to take part, consent forms were crossed through and then destroyed.

### Randomisation, concealment of allocation, and blinding

Randomisation to three sex-specific intervention groups in the ratio 1:1:1 was undertaken at the academic trial coordinating centre using individual-level randomisation achieved by means of a computer programme built into the trial database. This programme includes a minimisation algorithm to ensure that key prognostic characteristics are balanced across the trial arms at baseline (for example, new/repeat donor status, weight and age, as shown in Table 2). Randomisation was stratified by donation centre and gender. By the nature of the trial, participants have not been blinded to the intervention group to which they have been allocated.

**Table 2:** **Categories for minimisation variables**

	Men		Women	
**Age**	<50 years	≥50 years	<50 years	≥50 years
**Weight**	<70 kg	≥70 kg	<60 kg	≥60 kg
**Donor status**	New	Repeat	New	Repeat

### Baseline data collection

On the day following enrolment into the study, NHSBT confidentially retrieved data on participants from donor records, including donor number, donation number, donation date, email and mobile contact details, sex, month and year of birth, NHS number and previous donation and deferral history in the past 5 years. Using secure systems, these data were transferred to the academic coordinating centre to facilitate contact of participants, implementation of trial protocols, tracking of participants’ appointments and attendances throughout the trial. A few days after joining the trial, participants received two emails: one containing a uniform resource locator (URL) link and requesting completion of the trial’s baseline questionnaire, and another containing a password to access the online questionnaire. Participants’ trial IDs were embedded within the URL link using a custom tag to anonymously track completion of the questionnaire. Participants were requested to complete the questionnaire within 7 days and received two email reminders and a phone call between days 7 and 14 if they did not respond. The baseline questionnaire has been designed to take approximately 15 minutes to complete and includes the following:

1. Compulsory questions including month and year of birth, sex, height, and weight. This information was used to verify that the questionnaire had been completed by the individual to whom it was sent, and also for minimisation algorithm described above.
2. Quality of life questions, using the Short Form Health Survey (SF-36v2) questionnaire. The SF-36v2 is a generic measure of health status that has been reported to differentiate well between health benefits produced by a range of treatments irrespective of age, disease condition, or treatment group [17–20]. It is composed of 36 questions and produces an eight-scale profile of functional health and well-being, as well as two psychometrically-based summary measures (that is a physical component score (PCS) and mental component score) and a preference-based health utility index.
3. Previous history of iron deficiency, which may relate to susceptibility to iron deficiency.
4. Brief lifestyle information including, diet (particularly related to iron intake and absorption), alcohol intake, smoking and physical activity.

Only participants who returned the baseline questionnaire were eligible for randomisation. After randomisation, participants were advised of their new inter-donation interval by email and were also informed that appointments should be made through NHSBT’s ISAT team. The ISAT team was responsible for making this first appointment to coincide with the participant’s allocated donation frequency. For subsequent appointments during the trial, donors are given the option, and are encouraged, to make all appointments at their selected donor centre on the occasion of future donation visits.

Once randomised, donors’ participation in the trial was flagged on their donor record together with their allocated donation frequency. This flag is critical to (1) NHSBT donor centre staff, since it is essential for the booking of future appointments at the correct interval; (2) the academic coordinating centre’s helpdesk and the ISAT team, since the flag helps to identify donors’ status on the trial and resolve any queries; and (3) data management processes, such as retrieving data on donors at regular time-points from NHSBT’s donor database and securely transferring this information to the trial coordinating centre for the purposes of monitoring appointment bookings and attendance, deferrals, adverse events of donation, positive microbiology results and deaths.

### Blood sample collection, processing, and analysis

At the baseline donation visit at which the participant’s eligibility was assessed and consent obtained, a research sample was taken from the satellite pouch that forms part of the routine blood collection unit (that is, a separate venipuncture is not required). The research sample was collected in three tubes: 3 ml EDTA, 6 ml EDTA and 6 ml serum, which were each inverted three times before placing in rigid boxes for transport to the three NHSBT sample holding sites at Manchester, Colindale (London) and Bristol. Samples were couriered from the holding sites to the central study laboratory (UK Biocentre, Stockport, UK) for processing in the morning following collection. The large majority of samples (97%) were processed within 24 hours. Samples were kept at ambient temperature from the point of collection until completion of processing. Fluctuations in temperature were monitored at various sites and time points during the collection period.

A full blood count was performed from the 3-ml EDTA blood sample using a Sysmex XN-2000 haematology analyser (Sysmex UK Limited, Milton Keynes, UK) to generate an extended profile of blood cell indices. Full blood count results of all participants were reviewed by the Haematology Review Group to identify clinically significant results for further consideration. Following centrifugation, the 6-ml EDTA sample was used to extract two 0.8-ml plasma aliquots and buffy coat, while the 6-ml serum sample yields two 0.8-ml aliquots. The plasma, serum and buffy coat aliquots are stored at -80°C prior to planned use (such as for serum ferritin analyses and DNA extraction from buffy coat). This sample collection protocol is being repeated in all participants at their last donation prior to completing their 2-year involvement in the trial. If donors are deferred or medically withdrawn from blood donation at the time their final research sample is due, they are being offered an appointment to give a research sample.

### Data collection between randomisation and the 2-year assessment

Participants are requested via email to complete an online questionnaire every 6 months during the trial follow-up period. The follow-up questionnaires collect information on the following:

1. Compulsory questions including month and year of birth to validate completion of the questionnaire by the individual to whom it is sent.
2. Quality of life using the SF-12v2. This abbreviated version of the SF-36v2 was chosen to reduce time burden on participants [21], as it takes only 5 minutes to complete. We are using information from this questionnaire to monitor participants’ self-rated health during the trial and provide a means to assess potential bias of trial outcomes due to selective attrition.
3. (Serious) adverse events ((S)AEs), which are assessed to monitor donor safety throughout their 2-year participation in the trial. Questions on (S)AEs are designed to take about 5 minutes to complete and include adverse events diagnosed/treated at hospital or by a doctor, diagnoses of low iron and symptoms associated iron deficiency (see Table 3).

**Table 3:** **(Serious) adverse events collected at 6-month intervals after randomisation, including serious adverse events, diagnoses and symptoms of low iron**

Type of adverse event	Description
**Data collected at 6, 12, 18 and 24 months**	
Serious adverse events, diagnosed/ treated at hospital or by a doctor	Heart problems including heart attack, stroke, mini-stroke, angina, heart failure
	Falls
	Transport accidents (when in charge of a vehicle)
	New illness
Diagnoses of low iron	Diagnosis of low haemoglobin by NHSBT
	Diagnosis of anaemia by general practitioner (GP) or hospital
	Prescription of iron supplements
Symptoms	Tiredness
	Dizziness
	Feeling faint
	Fainting
	Fit or seizure
	Breathlessness
	Palpitations
	Chest pain
	Restless legs [22]
***Additional data collected at 24 months***	
Symptoms	Severity of breathlessness [23]
	Severity of palpitations (that is, resulted in ECG?)
	Severity of chest pain (that is, resulted in ECG?)
	Headaches
	Sleep disturbances
	Irritability
	Reduced ability to concentrate
	Restlessness/inability to relax
	Pica

### Data collection at the 2-year assessment

On the anniversary of participants’ 2-year involvement in the trial, an email request is sent to participants requesting completion of a final 35-minute questionnaire which collects information on the following:

1. Month and year of birth as well as sex in order to validate the identity of the person completing the questionnaire.
2. Quality of life using the SF-36v2 questionnaire.
3. (Serious) adverse events. At this time-point an enhanced set of questions ask about a broader range of symptoms and the severity of selected symptoms (see Table 3).
4. Medication and supplement use, including glucose-lowering, antihypertensive, and lipid-lowering drugs as well as use of over-the-counter dietary or vitamin supplements (with a specific additional question on whether these supplements contain iron).
5. Cognitive function tests, including Stroop Test (attention and reaction times), Trail Making Test (executive function), Pairs Test (Episodic Memory), and Reasoning Tests (intelligence). These tests, which have been adapted from the Cardiff Cognitive Battery, have been specifically designed for cognitive testing in epidemiological settings [24]. The tests are preceded by a five-item mood questionnaire to adjust for effects that may skew results of cognitive function [25, 26].
6. Donor beliefs and ideas about blood donation, and in particular whether increased frequency of donation raises specific difficulties or concerns; these questionnaire data will be augmented by a small number of in-depth interviews.
7. Invitation to take part in physical activity monitoring.
8. Recent physical activity questionnaire (RPAQ) which includes questions about physical activity during the past 4 weeks across four domains (leisure time, occupation, commuting and domestic life) [27, 28].

The administration and reminder procedures for the baseline, interim, and final questionnaires have been identical.

### Physical activity monitoring

As part of the 2-year assessment, all participants will be invited to take part in physical activity monitoring using the tri-axial accelerometer AX3 (Axivity, York, UK) to measure the impact of more frequent blood donations on activity levels. Donors who respond positively will be randomly selected to take part, with a target of 1,000 participants in each of the six sex-specific randomised intervention groups.

### Cost effectiveness analysis

A full economic analysis of alternative strategies for maintaining the supply of blood to the NHS is planned. INTERVAL trial data will be used to estimate the effect of reduced minimum inter-donation intervals on the number of blood donations made, donor’s quality of life, deferral and costs. A discrete event simulation model will be developed to extrapolate the cost effectiveness of alternative minimum donation intervals over 10 years. Data from NHSBT’s donor register (the PULSE database, Savant, Burton-in-Kendal, UK) will be accessed to provide donor characteristics and donation history to define the target population, and to estimate long-term rates of donation, under current minimum recall intervals, and characteristics of the blood collection service. Discrete choice experiments will be conducted to investigate donor willingness to donate blood under future changes to the blood collection service. The analysis will use the findings from the discrete choice experiments, together with those from the re-analyses of INTERVAL and PULSE data, to estimate the cost-effectiveness of alternative blood collection strategies over a 10-year time horizon for general populations of whole blood donors.

### Outcome measures

#### Primary outcome

The primary outcome is the number of blood donations made over 2 years. The number of blood donations up to (and not including) the 2-year anniversary date of recruitment will be counted.

#### Key secondary outcome

The key secondary outcome is physical well-being at 2 years, derived from the PCS of the SF-36v2 [21].

#### Other secondary outcomes

Other secondary outcomes, also to be assessed at 2 years, will include the following:

i) The number of ‘deferrals’ of donors over the 2 years of the trial due to low haemoglobin levels and/or other factors (for example, clinical reasons, tattoos, piercings, and foreign travel).
ii) Measures of iron status, including serum ferritin and reticulocyte haemoglobin.
iii) Cognitive function tests of attention, executive function, intelligence, and episodic memory.
iv) Physical activity measured by accelerometry.
v) Cost effectiveness.
vi) Donor attitudes assessed through questionnaire responses and interviews.

### Sample size

#### General considerations

The trial’s sample size was determined on the basis of partially overlapping considerations, including NHSBT's duty of care to 1.4 million blood donors per year (making it vital for the service to ascertain even subtle changes in health and well-being related to blood donation) and the need to generate sufficiently compelling evidence to influence regulators and policy-makers, since substantial benefits to the blood supply (and value for money for NHSBT) could accrue with even a comparatively small, but definite, increase in blood donation rates. As noted above, we have pre-specified interest in several subgroups because they seem *a priori* either more or less able to give blood on a more frequent basis. These donor groups are summarised in Table 4 together with their estimated prevalence based on data from previous studies [13, 29–31]. Furthermore, we will identify people in the top 20% for hereditary ‘robustness’ in relation to iron homeostasis/red cell indices by using genetic risk scores that summarise many genetic variants relevant to these traits.

**Table 4:** **Pre-specified subgroups with suspected higher or lower susceptibility to iron deficiency following blood donation**

	Estimated prevalence in National Health Service Blood and Transplant donor population
**More resilient to deferral and iron deficiency**	
Men	47%
Carriers of the human haemochromatosis (HFE) gene variants (C282Y and H63D)	27%
Women aged >50 y	20%
Serum ferritin levels above 95^th^ percentile	5%
**Less resilient to iron deficiency**	
Body mass index <22 kg/m^2^	14%
New blood donors	10%

#### Sample size calculations related to the outcomes

This trial has been powered on its primary endpoint (number of blood donations over 2 years) and key secondary endpoint (physical component quality of life score).

(1) *Number of blood donations:* As the men in this trial are assigned to 12-wk versus 10-wk versus 8-wk inter-donation intervals, the maximum number of donations possible over the 2-year trial duration are 8, 10, and 12 donations, respectively (that is, a 25% proportional increase in donation rates when comparing a 10-wk versus 12-wk frequency, or a 50% proportional increase when comparing a 8-wk versus 12-wk frequency). Women are assigned to 16-wk versus 14-wk versus 12-wk inter-donation intervals, corresponding to a maximum number of donations over 2 years of 6, 7, and 8 respectively. However, when considering the current nonattendance rates of donors, such maximal differences based on more frequent donation intervals are unlikely to be achieved in practice.

**Figure 2:**
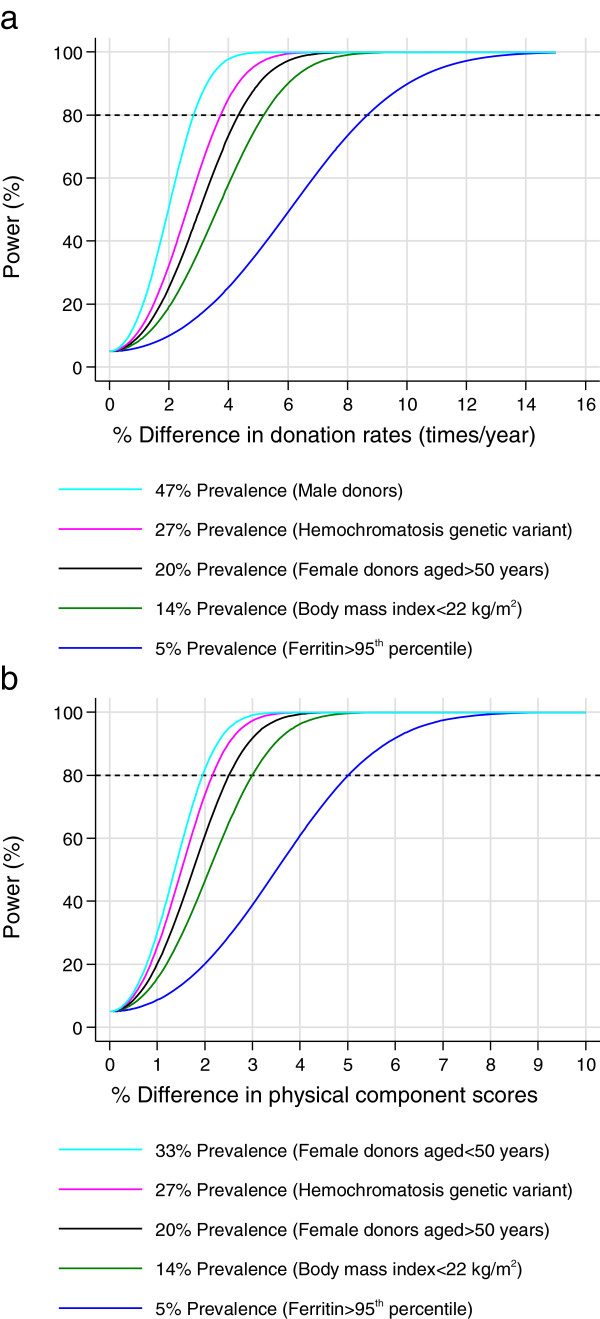
**Power to detect differences in the primary and key secondary outcomes. a**: Power to detect difference in donation rates. **b**: Power to detect difference in physical component score of SF-36 survey.

Hence, the power calculations are based on having 80% power to detect a more realistic 5% increase in the number of donations over two years. Such an increase would be of relevance to NHSBT since it would yield an additional 70,000 units of blood per year from the same donor base. Figure 2a shows that 50,000 trial participants will provide 80% power to detect ≥5% difference in donation rates/year in subgroups with a prevalence of ≥10% (at least 5,000 donors). The sample size calculation assumes type I error = 0.05 and mean donation rate = 1.6 times/yr in the standard donation frequency group (control), and between subject SD = 0.7 times/yr in each group.

(2)*Quality of life:* After making allowances for up to one third of participants not completing the final questionnaire, Figure 2b shows that 50,000 trial participants will provide 80% power to detect ≥3% mean difference in the PCS of the SF-36 in subgroups with a prevalence of ≥10%. This assumes a type I error = 0.05 and PCS = 50 in the standard donation frequency group (control), and between subject SD = 10 in each group. As a comparative benchmark, Patterson [32] showed a 3% (1.5-point) difference in the SF-36 PCS in women who self-reported iron deficiency in the past two years compared with those who had no such history.

### Data analysis

#### Principles

Intention-to-treat analyses will be used, comparing groups as randomised. Results for men and women will be analysed separately. The principal pair-wise comparisons of groups for men will be between 10-wk and 12-wk, and between 8-wk and 12-wk intervals. For women, these comparisons will be between 14-wk and 16-wk, and between 12-wk and 16-wk intervals. For outcomes assessed at both baseline and at the 2-year follow-up, analyses of the 2-year follow-up measures will be adjusted for the baseline measures. Other baseline characteristics (age, centre, BMI and weight, SF-36v2 physical and mental component scores, haemoglobin and ferritin) will be adjusted for in secondary analyses. Tests of interaction will assess whether results differ between the subgroups pre-specified above. Multiple testing will be taken into account when interpreting these results. The trial will be reported according to CONSORT guidelines.

#### End-of-trial analyses

The total blood collected by 2 years will be expressed in units (470 ml) per person per year. Means will be compared between groups using a t-test, with subsidiary analyses of covariance adjusting for baseline characteristics. The mean differences between groups in the PCS of the SF-36v2 will be adjusted for baseline PCS by analysis of covariance, and in secondary analyses for other baseline characteristics. Possible bias from missing data will be investigated by multiple imputation within each randomised group based on sequential additions to the imputation model: (1) baseline characteristics, (2) interim SF-12v2 scores, (3) deferral at previous blood collections, (4) number of blood collections made, and (5) number of reminders to elicit web questionnaire responses [33–35]. Other secondary outcomes will be analysed similarly. The proportion of deferrals will be analysed using logistic regression, allowing for potential over-dispersion between individuals. The baseline predictors (including genetic characteristics) of levels of haemoglobin and ferritin, and rates of deferral will be investigated using multiple (logistic) regression, employing cross-validation techniques to prevent over-fitting [36].

### Ethical and safety issues

The trial has received ethics committee approval from the National Research Ethics Service Committee East of England - Cambridge East (Research Ethics Committee (REC) reference 11/EE/0538). It is important to note that the features used to ensure the safety of donors in routine blood donation in NHSBT are being used to ensure the safety of donors in the INTERVAL trial.

The role of the trial’s Independent Data Monitoring Committee is to provide independent advice, based on un-blinded analysis of data, to the Trial Steering Committee in order to ensure the safety of the participants. Hence, at least every 6 months, the trial’s Independent Data Monitoring Committee reviews un-blinded safety data provided by the trial’s independent statistician. These results derive from the various sources described above, including the SF-12v2 questionnaire data and the self-reported adverse events questionnaire.

An increase in deferrals or adverse events compared to habitual donation patterns may prompt participants to request withdrawal from the trial. Participants are free to withdraw from the trial at any time without giving reasons. However, in order to maintain maximal data capture participants are asked to continue to complete the trial questionnaires without donating blood. If participants still wish to withdraw from the trial, then they are given the option to allow permission to the research team to retain and use data already collected from them. Participants are free to opt out of this permission.

### Patient and public involvement

Donors have been involved in the design and undertaking of the INTERVAL trial in several ways, such as through (1) provision of feedback during the pilot study preceding this trial (that is, the Cambridge CardioResource Study) via questionnaires, interviews, and helpline services; (2) review of research proposals and questionnaires; and (3) membership on the Trial Steering Committee.

### Trial oversight

In addition to the Independent Data Monitoring Committee and the Research Ethics Committee mentioned above, INTERVAL benefits from the input of other trial oversight committees. The Trial Steering Committee (which includes several senior clinical and academic members who are independent from the trial investigators, a lay representative, and representatives of various stakeholders in INTERVAL) monitors the overall conduct of the trial and, through its independent Chair, provides strategic advice to the Trial Management Group. The Trial Management Group (which includes the investigators, trial coordinators, and operational staff from NHSBT) is responsible for overseeing day-to-day management of the study, liaising with NHSBT, and agreeing protocol amendments prior to submission to the research ethics committee.

## Discussion

To our knowledge, there are no previous or planned randomised trials in the UK or elsewhere that aim to define optimum inter-donation intervals and/or tailor such intervals to particular groups of blood donors. Although some randomised trials have been conducted or are in progress to evaluate the impact of oral iron supplementation [37, 38], they are complementary to INTERVAL and evaluate populations outside the UK. The INTERVAL randomised trial is also complementary to recent major observational studies reported in blood donors, such as those in the US [39] and in continental Europe [40].

In summary, the experience so far of the INTERVAL trial suggests that it is possible to embed large-scale clinical research within NHSBT without disruption to routine services, and in a manner that is acceptable to donors. The INTERVAL trial will yield novel information about the effect of inter-donation intervals on donors’ physical and mental well-being as well as on blood services. The study will generate scientific evidence to help formulate blood collection policies in England and elsewhere.

## Trial status

Recruitment in INTERVAL was completed on 15 June 2014.

## Abbreviations

AEs: adverse events
BMI: body mass index
DNA: deoxyribonucleic acid
EDTA: ethylenediaminetetraacetic acid
HFE: human haemochromatosis gene
ID: identification
ISAT: INTERVAL study administration team
NHS: National Health Service
NHSBT: NHS Blood and Transplant
PCS: Physical Component Score
RPAQ: Recent Physical Activity Questionnaire
REC: research ethics committee
SAEs: serious adverse events
SF-12v2: Medical Outcomes Study 12-item Short-Form Health Survey, version 2
SF-36v2: Medical Outcomes Study 36-item Short-Form Health Survey, version 2
URL: uniform resource locator link.
